# The Removal of Atrazine and Benalaxyl by the Fly Ash Released from Kosovo A Power Plant

**DOI:** 10.1155/2022/9945199

**Published:** 2022-01-27

**Authors:** Esad Behrami, Kledi Xhaxhiu, Bedri Dragusha, Arianit Reka, Adelaida Andoni, Xhuljeta Hamiti, Spiro Drushku

**Affiliations:** ^1^Department of Chemistry, Faculty of Natural Sciences, University of Tirana, Tirana, Albania; ^2^Department of Pomology and Viticulture, Faculty of Agriculture and Veterinary, University of Prishtina, Pristina, Kosovo; ^3^Faculty of Natural Sciences and Mathematics, University of Tetovo, Ilinden n. n., 1200, Tetovo, North Macedonia

## Abstract

The development of low-cost adsorbent coal FA (Kosovo A) for pesticide removal is an important area of scientific research. With this study, we show the potential of adsorption of coal FA (Kosovo A) for the removal of benalaxyl and atrazine from water. We have found that the amount of adsorbed benalaxyl and atrazine increases with an increasing amount of coal FA (Kosovo A) in solution. The maximum capacity coal FA (Kosovo A) to adsorb benalaxyl and atrazine was found to be 0.46 and 0.45 mg/g according to the Freundlich equation and 3.48 and 3.33 mg/g according to the Langmuir equation. The Freundlich adsorption equation better explains the adsorption results of pesticides (benalaxyl and atrazine) in coal FA (Kosovo A), as the values of the recovery coefficient (*R*^2^) were higher in Freundlich equation than the Langmuir equation. The adsorption isotherms were of type L and show that the adsorption efficiency of the coal FA (Kosovo A) depends on the initial concentration of benalaxyl and atrazine in solution and the maximum removal of benalaxyl and atrazine was achieved at concentrations less than 10 *µ*g/ml. This study's results are expected to have implications for the use of coal FA (Kosovo A) for the removal of pesticides from water.

## 1. Introduction

Fly ash (FA), a relatively abundant and inexpensive material, is currently being investigated as an adsorbent for the removal of various organic pollutants from wastewater [1]. Although all the pollutant treatment techniques can be employed, they have their inherent advantages and limitations. Among all these methods, adsorption process is considered better than other methods because of convenience, easy operation, and simplicity of design. A fundamentally important characteristic of good adsorbents is their high porosity and consequent larger surface area with more specific adsorption sites. This paper presents a review of adsorption of different pollutants using activated carbon prepared from FA sources and the attendant environmental implications. Also, the ways of overcoming barriers to fly FA utilization together with regeneration studies are also discussed. Regeneration is a very important aspect of the adsorption from economic and environmental point of view. It actually answers various questions and anxieties as to what would happen to the adsorbent after adsorption in order not to render the whole findings useless since such adsorbent may contain toxic adsorbates [2]. Fly ash is a type of industrial waste that can cause multiple environmental problems if discharged into the air. On the other hand, because of its high porosity, large specific surface area, and other unique characteristics, FA can also be used as a low-cost and high efficient adsorbent for the treatment of environment pollutants [3]. The utilization of FA from coal burning power plants to remove pollutants from aqueous solutions has also been studied by several authors. Ugurlu and Salman achieved over 96% efficiency for phosphorous removal in batch and continuous conditions. Aksu and Yener compared sorption capacity of FA and GAC towards o- and p-chlorophenols, concluding that the alternative sorbents may constitute an interesting alternative, once the difference of sorption capacity (for GAC, 380 and 422 mg/g, and for FA, 98.7 and 118.6 mg/g, for o-chlorophenol and p-chlorophenol, respectively) is not significant when compared with the cost of the sorbent [4]. Atrazine (2-chloro-4-ethylamino-6-isopropylamino-1, 3, 5- triazine), a chlorinated triazine herbicide, is one of the most widely used pesticides in crops such as maize, sugarcane, citrus fruits, sorghum, and pineapple and has been sold under different commercial names such as Aatrex, Aatratol, Bicep, and Gasaprim. Due to its extensive use, long life, high geochemical mobility, and various toxic properties (LD50 1,869–3,080), it has environmental significance and needs remediation [5].

## 2. Materials and Methods

### 2.1. The Adsorbent

Coal FA (Kosovo A) was collected at the (Kosova A) PP, Bardhi i Madh 42° 38′ 13″ North, 21° 1′ 34″ East, altitude 554 m, and the coal is mainly lignite.

**Figure 1:**
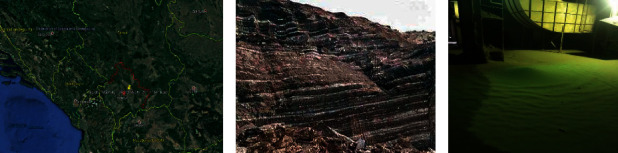
(a) Map of the Republic of Kosovo, (b) lignite layers, and (c) photo of coal FA (Kosovo A).

In Figure 1(a), we have presented the map of the Republic of Kosovo, specifically, the coordinates of surface coal mining in the place called Bardhi i Madh. In Figure 1(b), we have the appearance of surface mining and in Figure 1(c), we have the appearance of the FA sampling site of the (Kosova A).

In the Table 1, we have presented the FA data of the (Kosova A) *P*, data which were obtained from the characterization of a sample by the X-ray diffraction method.

**Figure 2:**
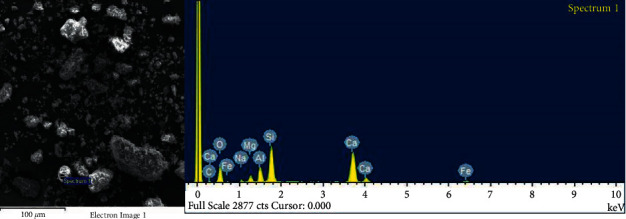
Semiquantitative analysis of FA power plant (Kosovo A) with SEM-EDX method.

In Table 2, we have the data in % of the components of the fly ash of the coal power plant Kosova A, where it is seen that the oxygen element dominates followed by Ca, Si, C, and Al. These data are also confirmed by spectrum 1 in Figure 2.

HF images of the power plant (Kosovo A) analyzed by using an scanning electron microscope (SEM) with resolution: 100 *µ*m; 2 × 20 *µ*m and 5 *µ*m.

**Figure 3:**
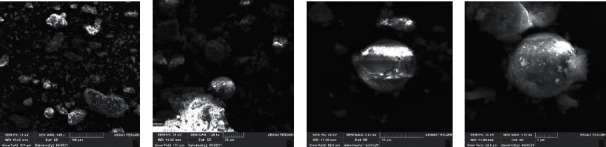
(a) Magnification 100 *µ*m; (b) magnification 20 *µ*m; (c) magnification 20 *µ*m; and (d) magnification 5 *µ*m.

Figure 3 shows the SEM images of the fly ash samples of the Kosova A power plant, where it is seen that the particles have a spherical shape with a contact surface considered, and for this, the HF of the Kosova A power plant can be considered as a good adsorbent of pesticides.

#### 2.1.1. Factors Affecting FA Adsorption Performance

Factors affecting adsorption performance are the effect of contact time and initial concentration of pollutants [2].

Table 3 shows the adsorption capacity of HF of coal in relation to organic compounds in general and to atrazine and benalaxyl in particular. The tabular data also include the values of the adsorbed amounts of these compounds which were obtained in this study.

### 2.2. Benalaxyl and Atrazine

#### 2.2.1. Benalaxyl

Benalaxyl is a broad-spectrum phenylamide fungicide. The common name is benalaxyl, while the IUPAC name is methyl N-phenylacetyl-N-2, 6-xylyl-DL-alaninate. The molecular formula of benalaxyl is C_20_H_23_NO_3_ with a molecular mass of 325.41 g mol^−1^ and has the following structure: https://pubchem.ncbi.nlm.nih.gov.

#### 2.2.2. Atrazin

Atrazine is a broad-spectrum herbicide. The common name is atrazine, while the IUPAC name is 6-chloro-N_2_-ethyl-N_4_-(propan-2-yl)-1, 3, 5-triazine-2,4-diamine. The molecular formula of atrazine is C_8_H_14_ClN_5_ with a molecular mass of 215.69 mol^−1^ and has the following structure: https://pubchem.ncbi.nlm.nih.gov.

**Figure 4:**
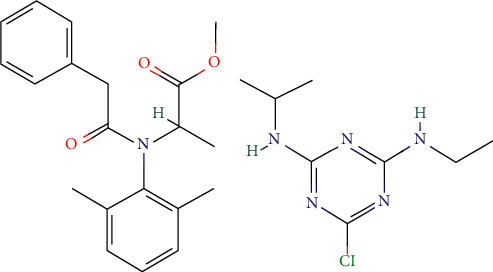
Structures of (a) benalaxyl and (b) atrazine.

Figure 4 shows the structures of pesticides: atrazine and benalaxyl which is the object of this research. Based on the steric effects (molecular packaging) of the structures of atrazine and Benalaxyl, it turns out that both pesticides show good adsorption affinity on the surface of the coal HF of the power plant Kosova A.

Pesticides are widely used in agriculture to increase crop yields and quality. Atrazine is one of the most widely used pesticides worldwide, being a long-term and large-area herbicide suitable for the removal of broadleaf weed species. Long-term atrazine residue accumulation in the soil accounts for 20%–70% of the applied dose during application and is one of the most commonly detected pesticides in soils and groundwater worldwide [14].

A number of studies explored modifying silicate minerals (clays and zeolites) as adsorbents for atrazine. examined removal of atrazine, lindane, and diazinone from water by organo-zeolites. However, the adsorption capacity for atrazine was the lowest (2.0 mmol/g) [15]. Atrazine can be mineralized by biological activity or immobilized by physicochemical processes, generating nonextractable residues; therefore, the conventional extraction methods for quantification of atrazine in soils may be inappropriate. As a result, the extraction method may be considered the most relevant step for quantification of atrazine and other triazines in soils; hence, it is of prime importance for the optimization of the extraction parameters. [16] Atrazine (ATZ) is one of the most heavily used types of herbicide that is currently widely applied in the agricultural operations of Western Australia. Therefore, the soil in this state is highly prone to exposure to atrazine due to agricultural operations such as storage, carrying, and application by pest control companies or users. [17].

#### 2.2.3. Adsorption Study

We have studied the kinetics of adsorption of benalaxyl and atrazine with physical and chemical properties in Table 4, in coal FAPP (Kosovo A) in a ratio of 0.5 g : 10 ml (0.5 g of coal FAPP (Kosovo A) with 10 ml of distilled H_2_O), where we have obtained aqueous solution of benalaxyl and atrazine (10 g/ml) in time 10, 20, 30, 60, 120, and 360 min and 24 h. We held a blind test to observe any adsorption of benalaxyl and atrazine on the glass surface or their degradation during equilibrium. After reaching equilibrium, the flight coal FAPP (Kosovo A) was centrifuged at 1500 rpm for 20 min, and then we characterized benalaxyl and atrazine from the supernatant with GC/MS-QP2010S. The adsorbed amounts of benalaxyl and atrazine from coal FAPP (Kosovo A) was calculated from the changes of initial and final concentrations in the supernatant. We have observed that there is no adsorption process of benalaxyl and atrazine during equilibration time. To study the effect of the amount of coal FAPP (Kosovo A) on the adsorption of benalaxyl and atrazine, different amounts of coal FAPP (Kosovo A) (0.1–2 g) were equilibrated with 10 ml of aqueous solution of benalaxyl and atrazine (10 *µ*g/ml) for 2 hours. A blind test, without coal FAPP (Kosovo A), was held as a control. After equilibration, the coal FAPP (Kosovo A) suspension was centrifuged at 1500 rpm for 20 min, and the benalaxyl and atrazine residues were characterized in supernatant with GC/MS-QP2010S. The adsorbed amounts of benalaxyl and atrazine from the coal FAPP (Kosovo A) were calculated as mentioned above (from the extract). To obtain adsorption isotherms for benalaxyl and atrazine in coal FAPP (Kosovo A), we have 1.0 g for benalaxyl and 0.2 g for atrazine and 10 ml of aqueous solution of benalaxyl and atrazine in a 50 ml sample which were balanced for 2 h in room. A blind test, without coal FAPP (Kosovo A), was kept as a control. In these solutions, the concentrations of benalaxyl and atrazine varied between 2.5 and 500 *µ*g/ml for benalaxylin and 2.0 and 10 *µ*g/ml for atrazine, and each concentration was repeated three times. After equilibration, the coal FAPP (Kosovo A) suspension was centrifuged at 1500 rpm for 20 min, and the benalaxyl and atrazine residues were characterized by the supernatant (extract) with GC/MS-QP2010S. We have calculated the adsorbed amounts of benalaxyl and atrazine residues from coal FAPP (Kosovo A) as mentioned above. The desorption of benalaxyl and atrazine from coal FAPP (Kosovo A) has been studied in the same tests, where after adsorption, we have selected only two concentrations for desorption. After studying the adsorption, 5 ml of the supernatant was removed and 5 ml of fresh distilled water was added, and the suspension was balanced again for 2 hours. After reaching equilibrium, we suspended the suspension of the coal FAPP (Kosovo A) at 1500 rpm for 20 minutes, decanted the supernatant, and characterized the residues of benalaxyl and atrazine as supernatant. Coal FAPP (Kosovo A) obtained after centrifugation has undergone two more desorption cycles. We performed a total of three desorptions for each sample and calculated the total amount desorbed by adding the amounts of benalaxyl and atrazine absorbed during each desorption.

#### 2.2.4. Extraction and Analysis

Benalaxyl and atrazine (Figure 2) residues in water samples were extracted by shaking the samples for 15 min (20 ml) with hexane + dichloromethane 1 : 3 (5 ml). The samples were then allowed to stand for 15 min, and 1 g of sodium anhydride sulphate was added to each test to remove any traces of moisture from the samples [18]. Benalaxyl and atrazine residues in the extraction solvent (hexane + dichloromethane 1 : 3) were analyzed by GC/MS-QP2010S.

## 3. Results and Discussion

**Figure 5:**
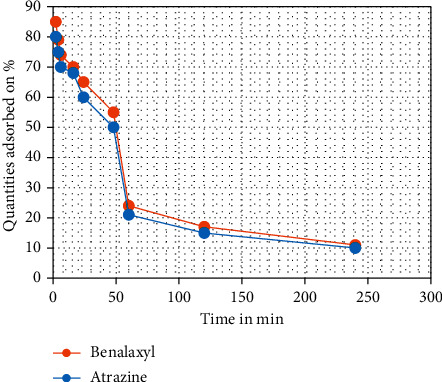
The % adsorbed amount of benalaxyl and atrazine in coal FAPP (Kosovo A) over time.

The results of the kinetics of benalaxyl and atrazine show that their adsorption in the coal FAPP (Kosovo A) is very fast, where 80% of the amounts of benalaxyl and atrazine are adsorbed in the coal FAPP (Kosovo A) in the first 10 minutes (Figure 5)

**Figure 6:**
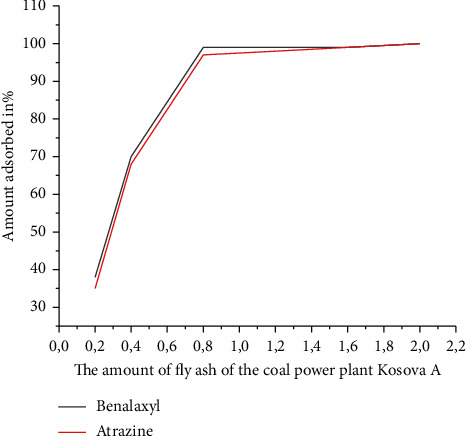
Amount of adsorbed % of benalaxyl and atrazine in coal FAPP (Kosovo A) depending on the amount of coal FAPP (Kosovo A).

Freundlich model is applicable for multilayer adsorption, while Langmuir isotherm model is for monolayer adsorption [19]. With the gradual developments of diverse adsorbent materials, the field of adsorption has become broader and specific in nature for particular pollutants including heavy metals, phenols, antibiotics, and pesticides [20]. We did not notice any significant change in the adsorption of benalaxyl and atrazine from the coal FAPP (Kosovo A) after 120 min. For this reason, we have chosen the time 2 h as the equilibrium time. [21, 22]. We have also noticed that coal FAPP (Kosovo A) has the maximum adsorption capacity for benalaxylin, followed by atrazine. The effect of the amount of coal FAPP (Kosovo A) on the adsorption of atrazine and benalaxyl is shown in Figure 6 [23]. The results of this experiment show that the adsorbed amounts of benalaxyl and atrazine in coal FAPP (Kosovo A) increased with increasing amount of coal FAPP (Kosovo A). Atrazine adsorption was almost 100% when the amount of coal FAPP (Kosovo A) in solution (10 ml) was 0.5 g. The amount of FA required for 100% adsorption of benalaxyl was 0.45 g per 10 ml of solution [2].

**Figure 7:**
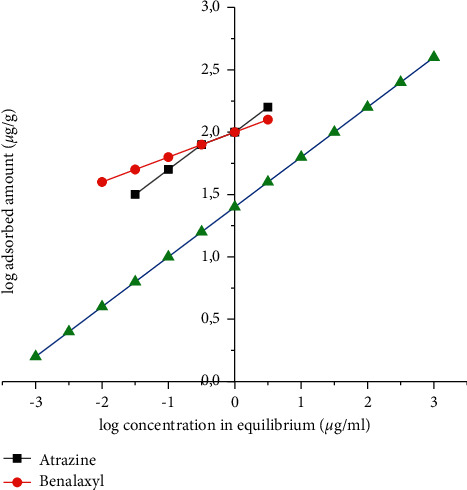
Freundlich adsorption isotherm for benalaxyl and atrazine in coal FAPP (Kosovo A).

Adsorption data for benalaxyl and atrazine in coal FAPP (Kosovo A) were found from the Freundlich adsorption equation:

(1) $$\begin{array}{c} \log\;C_{\text{ads}}=\log\;K_{f}+\frac{1}{n}\log\;C_{e}, \end{array}$$

where *C*_ads_ is the adsorbed amount of pesticides in equilibrium (*µ*g/g), *C* is the concentration of pesticides in equilibrium (*µ*g/ml), and *K*_*f*_ and 1/*n* are constant. Freundlich *K*_*f*_ constant (intercept) represents the amount of pollutant adsorbed at an equilibrium concentration of 1 *µ*g/ml. The constant 1/*n* (slope) is the measure of the adsorption intensity and reflects the degree to which the adsorption is a function of the pollutant concentration (Figure 7 and Table 5). [24] The values of the correlation coefficient for all cases were very high (*R*^2^ > 0.99), indicating that the Freundlich adsorption equation satisfactorily explained the adsorption results of benalaxyl and atrazine in coal FAPP (Kosovo A), and the results were significant at 99%. Earlier, it was shown that compared to the Langmuir adsorption equation, the Freundlich adsorption equation better explained the atrazine adsorption results in flight ash [25].

The slope values (1/*n*) for benalaxyl and atrazine in the adsorption process from the slope values (1/*n*) for benalaxyl and atrazine in the adsorption process from the coal FAPP (Kosovo A) were <1 suggesting isotherms of nonlinear adsorption. Slope value <1 indicates *L*-type isotherms, which are characterized by decreased adsorption at higher aqueous concentrations of the compounds, i.e., adsorption of benalaxyl and atrazine in the coal FAPP (Kosovo A) was concentration dependent which was <1 suggesting isotherms of nonlinear adsorption. Slope value <1 indicates *L*-type isotherms, which are characterized by decreased adsorption at higher aqueous concentrations of the compounds, i.e., adsorption of benalaxyl and atrazine in the coal FAPP (Kosovo A) was concentration-dependent [21, 26]. This type of adsorption isotherms is observed when the molecules are in a flat position, not experiencing strong competition from the water molecule, which explains the high affinity to the adsorbent for the solution at low concentrations [27]. However, if the concentration increases, the adsorbent sites become restrictive; therefore, the adsorption decreases [28]. Earlier, Konstantinou and Albanis [21] and Majumdar and Singh [29] reported *L*-type adsorption isotherms for the adsorption of pesticides into fly ash and soil mixtures.

Comparison of *K*_*f*_ values for benalaxyl and atrazine showed that the adsorption capacity of coal FAPP (Kosovo A) for benalaxyl is 0.47 mg/g followed by atrazine (0.41 mg/g). [30].

The order of adsorption of benalaxyl and atrazine in coal FAPP (Kosovo A) can be explained by their solubility in water because their adsorption is inversely proportional to their solubility in water [31, 32]. The solutions of benalaxyl and atrazine in water are 28.6 mg/L at 20°C and 33.0 mg/L at 25°C. Thus, benalaxyl, which has the lowest solubility in water, is more adsorbed to the coal FAPP (Kosovo A) [33].

**Figure 8:**
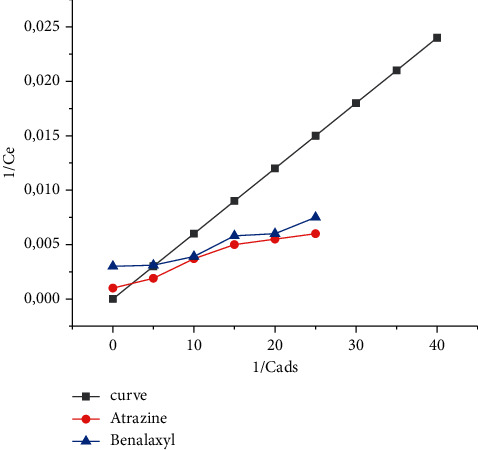
Langmuir adsorption isotherm for benalaxyl and atrazine in coal FAPP (Kosovo A).

Furthermore, the adsorption data were analyzed with the Langmuir equation:

(2) $$\begin{array}{c} \frac{1}{C_{\text{ads}}}=\frac{1}{q_{m}}+\frac{1}{q_{m}}bC_{e}, \end{array}$$

where *q*_*m*_ is the Langmuir constant which represents the maximum capacity of a layer and *b* is the Langmuir constant associated with the adsorption energy [34]. The ratio between 1/*C*_ads_ and 1/*C*_*e*_ for pesticide adsorption is shown in Figure 8. The values *q*_*m*_ and *b* are estimated from the curve and slope and are given in Table 5. We have noticed that the monolayer capacity (*q*_*m*_) of coal FAPP (Kosovo A) for pesticides (*µ*g/g) was increasing in the order: atrazine < benalaxyl, showing that coal FAPP (Kosovo A) has a maximum capacity to adsorb benalaxyl (3.48 mg/g) and atrazine (3.33 mg/g) [24].

This is similar to the results for the adsorption of benalaxyl and atrazine found from the Freundlich adsorption isotherm; however, the values found for the adsorption capacity of benalaxyl and atrazine using the Langmuir equation are almost 7 times higher than the values found using the equation Friendly [32]. Furthermore, parameter *b*, which is constant for the adsorption process and reflects the affinity of the adsorbent for pesticides, showed that the coal FAPP (Kosovo A) has maximum affinity for benalaxyl and atrazine [35].

This finding is consistent with the previous results whose results showed that the coal FAPP (Kosovo A) has a high affinity for both benalaxyl and atrazine [29].

Also, the value of the recovery coefficient (*R*^2^) for the adsorption of benalaxyl and atrazine using the Langmuir isotherm is 0.95, which is lower than the corresponding values obtained using the Freundlich equation [36, 37]. Furthermore, the results of the second experiment showed that the coal FAPP (Kosovo A) has a maximum holding capacity for benalaxyl followed by atrazine. Therefore, based on the above data, it can be seen that Freundlich isotherm best explains the adsorption results of benalaxyl and atrazine coal FAPP (Kosovo A) because the value of the recovery coefficient (*R*^2^) for adsorption was very significant (level 99%) [1, 38].

Comparing the efficiency of pesticide removal from water with adsorbent (coal FAPP (Kosovo A)) with other low cost adsorbents used by previous researchers categorizes it among the five efficient and low cost adsorbents [39], i.e., charcoal, rubber granules, bottom ash, macrofungus Sajor Caju, and Florida to remove atrazine from drinking water [40, 41]. Charcoal shows the best adsorption capacity of atrazine with *Q*_max_ 0.80 mg/g followed by rubber granules with 0.47 mg/g, and then the maximum capacity of coal FAPP (Kosovo A) to adsorb benalaxyl and atrazine was found to be 0.46 and 0.45 mg/g, according to the Freundlich equation [24, 42]. Thus, the result of our study suggests that coal FAPP (Kosovo A) has a good capacity to remove pesticides from aqueous solution and at low concentrations of pesticides (<10 *µ*g/ml) can remove more than 99% of them [35, 36].

Results in Table 6 indicate that desorption of the sorbed compounds from fly ash of the coal of power plant (Kosovo A) was concentration dependent. Lesser amounts of herbicides were desorbed from the coal FAPP (Kosovo A) when sorption was carried out at low concentrations. Only 10.7% of sorbed benalaxyl was released after 3 repeated desorptions from coal FAPP (Kosovo A) when sorption was carried out at 10 *µ*g/ml concentration. Desorption of atrazine was studied also at 10 *µ*g/ml concentration, and during 3 successive desorptions, nearly 8.9% of the sorbed atrazine was desorbed. These results suggest that if we compare desorption of benalaxyl and atrazine from coal FAPP (Kosovo A) at 10 *µ*g/ml concentration, benalaxyl is the maximum desorbed.

## 4. Conclusion

In more analyzes performed, we were convinced that the internal standard should be added to the organic extract separated from the aqueous solution. The best extractor is the mixture: Heksan + dichloromethane in a ratio of 3 : 1 by volume, the extraction is done with the minimum possible volume of solvent, so that the final volume is as small as possible and we have a concentration that is normally analyzed in the gas chromatograph. Studying the dependence of the adsorption of benalaxyl and atrazine from coal FAPP (Kosovo A) on aqueous solutions, from the time of contact coal FAPP (Kosovo A) + water, it is noticed that, from 120 min to 180 min, there is no visible adsorption of benalaxyl and atrazine on coal FAPP (Kosovo A). Further studies were therefore extended at intervals from 10 min to 120 min. To stabilize the analysis on the gas chromatograph, a series of determinations were performed with standard solutions of benalaxyl and atrazine (99%) and the internal standard DBF (99.5%) until the achievement of optimal operating parameters, obtaining clear chromatograms with the respective points visible and easily calculated. We did not notice any significant change in the adsorption of benalaxyl and atrazine from the coal FAPP (Kosovo A) after 120 min. For this reason, we have chosen the time 2 h as the equilibrium time.

## Figures and Tables

**Table 1:** Data on coal FA (Kosovo A).

SiO_2_ (%)	CaO (%)	Fe_2_O_3_ (%)	Al_2_O_3_ (%)	MgO (%)	P_2_O_5_ (%)	TiO_2_ (%)	Na_2_O (%)	K_2_O (%)	V_2_O_5_ (%)	NiO (%)	SO_3_ (%)	MnO (%)	Others (%)
25.5	38.6	7.5	9.4	5.5	0.3	0.3	1.1	0.6	n.p	n.p	9.0	0.2	2.0

**Table 2:** Participation in % of elements in the fly ash of the Kosovo A power plant.

Element	Weight in %	Atom in %
C	8.69	13.83
O	53.17	63.52
Na	1.47	1.22
Mg	2.41	1.89
Al	4.88	3.46
Si	11.65	7.93
Ca	15.47	7.37
Fe	2.26	0.77
Total	100.00	

**Table 3:** Adsorption capacity of coal FA.

Organic pollutants	Fly ash type	Adsorption capacity	References
Atrazine	Coal FA	0.45 mg/g	This study
Benalaxyl	Coal FA	0.46 mg/g	This study
Atrazine	Coal FA	3.33 mg/g	This study
Benalaxyl	Coal FA	3.48 mg/g	This study
Atrazine	Coal FA	(1.2–11.6 *µ*g/g/50 *µ*g/g)	[5]
Cabofuran	Coal FA	1.54–1.65 mg/g	[6]
Phenol	Coal FA	67 mg/g	[7, 8]
2,4-Dichlorophenol	Coal FA	1.5–1.7 mg/g	[9]
2-Nitrophenol	Coal FA	5.80–6.44 mg/g	[10]
3-Nitrophenol	Coal FA	6.52–8.06 mg/g	[10]
4-Nitrophenol	Coal FA	7.80–9.68 mg/g	[10]
Cresol	Coal FA	85.4–96.4 mg/g	[11]
Carbofuran	Coal FA	1.54–1.65 mg/g	[6]
TCB	Coal FA	0.35 mg/g	[12]
HeCB	Coal FA	0.15 mg/g	[12]
Methylene blue	Coal FA	14.4 × 10^−5^ mol/g	[13]

**Table 4:** Physical properties of benalaxyl and atrazine.

Physical properties	Melting point	Relative density	Steam pressure	Description of physical condition and color	Solubility in organic solvents	Solubility of pure substance in H_2_O	Degree of hydrolysis at pH 4, 7, and 9
*Benalaxyl*							
Result	76.8°C	1.181 g/mL at 20°C	Extrapolating: 5.72 × 10-4 Pa 20°C 7.08 × 10-4 Pa 28°C 19.6 × 10-4 Pa 50°C	Pure active substance: white crystalline solid	(g/kg at 22°C) n-Heptane 19.4 xylene >250 Acetone >250 Ethylacetate >250 1, 2-Dichloroethane>250 Methanol >250	In distilled water: pH 6.1 28.6 mg/L at 20° C	The experimental half-lives at pH 9 were 86 days (25°C) and 157 days (20°C)
Reference	Costantini G. et al. 1995. Report no: 94/1087.C	Costantini G. etal. 1995. Report no: 94/1087.B	Costantini G. et al. 1995. Report no: 94/1087.B	Costantini G. et al. 1995. Report no:94/1087.B	Costantini G. et al. 1995 Report no: 94/1087.B	Costantini G. et al. 1995. Report no: 94/1087.B.	Masoero M., Crisippi T. 1982

*Atrazine*							
Result	177.0°C	1.2 g/cm^3^	40 × 10^−6^ Pa at 20°C	Colorless or white, odorless, crystalline powder	183 g/kg DMSO 52 g/kg chloroform 28 g/kg ethyl acetate 18 g/kg methanol 12 g/kg diethyl ether 0.36 g/kg pentane	33.0 mg/L at 25°C	The half-life of atrazine hydrolysis in distilled water at pH 3, pH 4.5 and pH 8 are: 373 days, 522 days and 657 days
Reference	Haynes, W.M. (ed.). CRC Handbook of Chemistry and Physics. 95th Edition. CRC Press LLC, Boca Raton: FL 2014-2015, p. 3–30	LO International Chemical Safety Cards (ICSC)	ILO International Chemical Safety Cards (ICSC)	NIOSH. NIOSH Pocket Guide to Chemical Hazards. Department of Health & Human Services, Centers for Disease Control & Prevention. National Institute for Occupational Safety & Health. DHHS (NIOSH) publication no. 2010-168 (2010). Available from: https://www.cdc.gov/niosh/npg.	Lide, D.R., G.W.A. Milne (eds.). Handbook of Data on Organic Compounds. Volume I. 3rd ed. CRC press, inc. Boca Raton, FL. 1994., p. V1: 323	Yalkowsky, S.H., he, Yan, Jain, P. Handbook of Aqueous Solubility Data Second Edition. CRC Press, Boca Raton, FL 2010, p. 152	J Environ Sci (China) 2001 Jan; 13(1):99–103

**Table 5:** Adsorption parameters for adsorption of benalaxyl and atrazine in coal FAPP (Kosovo A).

Pesticides	Freundlich constants			Langmuir constants		
	*K*_*f*_	1/*n*	*R* ^2^	*q*_*m*_	*b*	*R* ^2^
Benalaxyl	394.2	0.42	0,99	3489.6	388.9	0.95
Atrazine	379.4	0.38	0.99	3333.3	370.4	0.95

**Table 6:** Amount of benalaxyl and atrazine desorbed by coal FAPP (Kosovo A).

Concentration (*µ*g/ml)	The amount adsorbed	Amount desorbed (*µ*g/g)				Desorption in %
		I	II	III	Total	
Benalaxyl	460	36.1	6.4	4,3	46.8	10.17
10 *µ*g/ml						
Atrazine	450	32.5	4.9	3.0	40.4	8.9
10 *µ*g/ml						

## Data Availability

The reference data used to support the findings of this study are included in the article.
